# Some Factors in the Production of Epilepsy

**Published:** 1898-06

**Authors:** William House

**Affiliations:** Buffalo, N. Y., Late junior assistant physician Manhattan State Hospital, New York City.


### SOME FACTORS IN THE PRODUCTION OF EPILEPSY.

BY WILLIAM HOUSE, M. D., Buffalo, N. Y.,
Late junior assistant physician Manhattan State Hospital, New York City.

FOR centuries the falling sickness has been a subject to which physicians have devoted unremitting study and attention. The unfortunate character of the symptoms, the tendency in most cases to progressive increase in the frequency of the seizures, the associated impairment of the intellect, the unsatisfactory results from treatment and the gravity of the prognosis, combine to make it a most disagreeable affliction and a stumbling block in the path of progress and advance.

The only rational hope for improvement in our knowledge of this disease and consequently in our treatment, must be based upon a more complete mastery of the etiology, which can only be obtained by thorough study of the pathological changes that produce, accompany or result from epilepsy. Unfortunately our knowledge of the morbid anatomy of epilepsy is limited.

We are indebted in no small degree to Starr and Van Giesen, of New York, for elaborate descriptions of the changes found in the brains of a number of patients suffering from Jacksonian epilepsy. The cases were selected ones and were such as presented symptoms indicating local cerebral lesion, manifesting itself in the production of seizures involving only localised areas; all the cases recorded gave a history of trauma. With the aid of a surgeon the patients were trephined over the areas supposed to be involved and the brain searched for abnormalities, not only on the surface, but beneath it, by means of exploratory needles for evidences of increased density. In those cases in which distinct changes were found, the involved tissue was exsected and submitted to careful microscopical examination. Briefly, the results obtained were such as might naturally be expected to follow injury to cerebral tissue and consisted essentially in degenerative processes in the cells of the cortex, surrounding areas of more or less infiltrated scar tissue. The operations were followed in most cases by improvement or recovery, showing the causative relation of the morbid parts to the production of the epileptic seizure.

While the results of these and other observations on the morbid anatomy and treatment of Jacksonian epilepsy are of great interest and importance, they are unfortunately applicable to only a small proportion of the entire list of cases. Jacksonian epileptics differ from the vast majority of the sufferers from epilepsy, in that the manifestations are more or less localised, whereas in typical epilepsy the seizures are general and of much greater severity. Observations on Jacksonian epilepsy unquestionably aid in studying the disease, no matter what its type, by furnishing an index or working basis and by properly directing the energies of the pathologists to whom we must look for aid in establishing a recognised etiology for the disease.

Causes of epilepsy, as given by the authors of text-books and monographs, vary greatly and include almost every imaginable disease-provoking element. Age, sex, heredity, alcoholism, syphilis (acquired or inherited) brain tumor, trauma, fright, digestive and renal disease, reflex disturbances, toxic factors, such as lead and uremic poisoning, these and numerous other causes are mentioned. Any of the above conditions may, no doubt, act in certain cases, either as predisposing or exciting, direct or contributing causes. The exact relation of some of them as trauma, tumor, syphilis and perhaps alcoholism, may easily be explained by the production of new growth, acting as a center of irritation, which under certain conditions produces the epileptic explosion. These pathological conditions are present in only a small percentage of cases, however, and if alone responsible for the convulsion, should produce, not the irregular spasmodic seizures, separated by prolonged intervals of quiet, but a single long-continued convulsion, or a series of shorter constantly recurring convulsions, separated by short intervals of quiet, the result of exhaustion, and reappearing in greater or less severity, according to the strength of the victim, until death results; such a condition actually occurs in status epilepticus. Nor do such lesions, producing only local convulsions, account for the complete loss of consciousness which occurs in every case of true motor epilepsy, lasting in time from seconds to hours and which associated with a convulsion not followed by paralysis, constitutes the pathognomonic symptom of this disease.

What, then, is the element directly productive of the epileptic attack? Some other factor existing as a concomitant feature, inconstant, present only part of the time and which when present produces the undue irritability of the nerve cells, or itself acts as the irritant agent, the "last straw" which causes the convulsion. Moreover, to account for the periodic nature of the seizure this element must be present, no matter whether there is gross pathological lesion, such as scar or gumma, or whether the brain is entirely free from such lesion, and it must be an element capable of variability in the intensity of its power to cause cerebral irritation, coming and going with the convulsion.

Van Giesen, director of the state pathological laboratory, with the unlimited material obtainable from the state hospitals for the insane and Craig colony for epileptics, is investigating with the aid of a large, well-equipped laboratory and skilled assistants, to prove a special toxicity of the blood at the time of the convulsion. His theory is on par with the theory of toxemia as the cause of insanity, and is probably a correct one, so far as it goes. It is distinctly corroborated in a clinical way, as evidenced by the greater frequency of convulsions in epileptics during periods of gastro-intestinal disturbances, notably constipation, which doubtless produces a mild degree of intestinal toxemia. Other observers are working along similar lines, but have not yet proved their theories.

A careful study of an attack of major epilepsy will bring forth symptoms bearing close resemblance to the seizures of apoplexy, paresis and chronic alcoholism. A typical attack of grand mal is usually preceded by indefinite sensations in various parts of the body, or by uneasiness. The patient may or may not recognise these sensations as auræ, although in the experience of the writer in an examination of a large number of cases at the Erie county hospital, at Buffalo, and the Manhattan state hospital, at New York, they were always present. The pulse prior to the attack is usually full and slow, the face flushed and congested. Excretory activity is diminished, the urine being scanty and high colored. The convulsion is ushered in with a cry and the patient falls unconscious with muscles set and rigid. The pulse remains full and of increased tension and is infrequent, often 50 or less per minute. For a moment the chest is fixed and rigid, the face congested or even cyanotic. The muscles now begin to twitch and the tetanic contraction gives place to spasms of a clonic nature. The eyes roll in their sockets, the respirations are slow and stertorous, the patient remains profoundly unconscious. By comparing the convulsion at this stage with the seizures of cerebral hemorrhage many points of resemblance are found. The loss of consciousness is duplicated by the syncope frequently observed in the early stages of a cerebral hemorrhage. The spasmodic muscular contractions are duplicated by the clonic spasms, occurring early in hemorrhage. In both conditions the respirations are at first slow and stertorous, pulse full and slow and pupillary reactions are also similar. It is true, that continuing, the cerebral hemorrhage results in a more or less complete paralysis of the muscles over which the involved centers preside. This has no analogus in epilepsy, because epilepsy stops before this point is reached. The stimulus which produces an epileptic seizure acts on the muscles as does a full medicinal dose of digitalis on the heart, *i. e.*, it causes increased activity while the stimulus of cerebral hemorrhage behaves as does a poisonous dose of digitalis on the heart and vasomotors, *i. e.*, paralysis results.

True epileptic seizures also bear marked resemblance to the convulsions occurring in alcoholics, in whom at post mortems we find edema of the brain, the so-called "wet brain" of alcoholics, a brain the cortex and membranes of which are distended—soft, edematous, dripping fluid on section, with ventricles dilated by fluid—a brain so enlarged that the cranial cavity seems too small to hold it. Equally similar to epilepsy are the convulsions seen in many paretics and which are spoken of as the epileptiform convulsions of general paresis. Without preceding knowledge of the case, it is often difficult to make a differential diagnosis between epilepsy and paresis in a patient seen for the first time during a convulsion. The writer has seen a paretic under close observation by men of wide experience in insanity, for a period of three months, during which he had several seizures before a diagnosis could be made. In necropsies, performed in similar cases in the experience of the writer, the examinations have revealed, in addition to the existence of cortical destruction and atrophy, always present in paresis, unusually marked distention of the membranes and subpial distention by fluid.

In all three of these latter conditions, post mortem examinations reveal evidence of increase in the fluid contents of the cranial cavity —an increase which causes pressure and which must therefore bear causal relation to the convulsion. The similarity of these convulsions to the seizures of epilepsy naturally leads to the inference that the convulsions depend on causes of a somewhat similar nature. As apoplectic and paretic convulsions result from pressure on the cerebrum, so also do epileptic attacks result from pressure and a careful study of a series of epileptic attacks lead to the belief that they are the result of a diffuse cerebral pressure, with perhaps some point or points of greater intensity, producing greater reaction of certain muscles; such areas being accounted for by the presence of often recognised lesions, such as scar or tumor.

Epileptic convulsions frequently cease with the passage of a quantity of urine, often pale, at times dark and concentrated. Urinary activity now becomes increased, the skin is covered with perspiration, the pulse becomes more rapid and softer, the slow, stertorous respirations give place to normal breathing, the pupils again react to light, the convulsive movements cease, the congested face gradually assumes a healthy tint, and the patient either slowly regains consciousness or falls into a deep heavy sleep from which he can be roused with difficulty. Many symptoms which are part and parcel of the convulsion point to a cerebral pressure which is general, while the method of the return to the normal state indicates equally relief from pressure, at times with great rapidity, more often slowly, just as might be expected to follow the quick absorption of fluid.

The post mortem examinations of patients suffering from grand mal reveals little that is recognised as characteristic. The brain in many cases shows no indication of abnormality and without knowledge of the events preceding death a diagnosis would often be impossible from the necropsy. In five examinations which the writer has seen on patients dying of status epilepticus, two at the Erie county hospital, at Buffalo, and three at the Manhattan state hospital, New York City, the only feature invariably present was an increase in the quantity of cerebro-spinal fluid. A careful examination of the records of over thirty necropsies performed at the latter institution, in which epilepsy was given either as a primary or contributing cause of death, shows that where the cerebro-spinal fluid has been mentioned at all an increase has nearly always been noted.

In four of the five cases mentioned no other change save apparent slight congestion was noted. In the fifth, seen at the Erie county hospital, in addition to the increased quantity of cerebro-spinal fluid, a band of fibrous tissue, apparently derived from the dura, was found dipping down beneath the end of a small convolution in the right occipital lobe, causing a very noticeable constriction.

The one feature always noted—that is doubtless the increased quantity of the cerebro-spinal fluid—is an important element in the production of epilepsy. Ever varying in amount, even in health, depending on the relative activity of general and lymphatic circulation, the cerebro-spinal fluid is sufficiently elastic and unstable in its movements to be readily affected by intrinsic and extrinsic factors. The ability of this fluid to cause convulsions is attested by Landois and Sterling, who state that "if it be suddenly withdrawn, convulsions or epilepsy may ensue, or, if it be rapidly increased in amount, coma may be produced." The American Text-book of Surgery says of spina bifida: "It may probably be reduced by gentle pressure or at least diminished in size, the diminution being attended with increased tension of the fontanelle and sometimes with stupor, convulsions or other nervous symptoms." Dr. Park, in his text-book, makes a similar statement. Pressure in spina bifida simply causes the fluid contents to force their way upward into the cranial cavity, thus increasing the quantity of fluid surrounding the brain, convulsions or coma resulting according to the degree of such pressure. The increase of this fluid in epilepsy is analogous to the edema in the alcoholic wet brain, to the increased quantity of fluid in paresis (which may be simply increased cerebro-spinal fluid or may result from the destruction of the cortical convolutions), and even to the more localised increase in quantity of fluid, occurring as a result of hemorrhage or abscess formation. All cause pressure and all tend to the production of symptoms bearing striking resemblance and differing principally in degree and extent.

What the exact condition may be that permits of such marked fluctuation as occurs in the quantity of this fluid in epilepsy is uncertain. Possibly a toxic element is responsible; more likely a defect in the activity of the lymphatic or excretory functions. It is certain that the thorough evacuation of the intestinal tract in epileptics, especially if hydragogue cathartics are used, often prevents a convulsion, and care in this respect materially diminishes the frequency of the seizures. Convulsions in habitual epileptics are augmented in frequency and in severity by failure to attend to this detail. Moreover, the naturally sluggish organs of the average epileptic predisposes to excretory inactivity. Whether the unloading of the bowels exerts a purely derivative effect, or whether it prevents absorption by the removal of toxic elements, or both, is open to question.

Heredity is conceded by all authorities to play an important rôle in epilepsy—not the direct transmission of the disease, but the transmission of a brain particularly susceptible to irritation. Aside from the cases giving distinct family histories of neuroses of various sorts, the parents of epileptics will often be found of a very low order of intelligence. They are stolid, dull-witted, unstable, the subjects of vicious habits, without discoverable morals, appear to suffer from defective nutrition and procreate idiots, imbeciles, criminals or epileptics. The writer has particularly observed such cases among the relatives visiting the epileptic insane of the Manhattan state hospital. Of all visitors the blood relations of epileptics are the most stupid, unreasonable and disagreeable with whom, as a class, dispensary and hospital physicians have to deal. It is probable that the same conditions which predispose to an irritable and unstable cerebral cortex gives rise also to a defective circulatory and lymphatic activity.

In terminating, the writer would invite attention to the following conclusions: (1) While we may have a definite focus of irritation, as in Jacksonian epilepsy, this focus is alone scarcely sufficient to account for all the symptoms which together constitute a true attack of grand mal. (2) No such focus is discoverable in the majority of cases and the post mortem examination reveals little evidence of cerebral disease that can be considered as characteristic. (3) The one feature most often seen in patients dying of status epilepticus is an increase in the quantity of cerebro-spinal fluid. (4) This fluid, fully capable of producing pressure, must be regarded as an important factor in the etiology of epilepsy. (5) The increase in the quantity of cerebro-spinal fluid is probably more or less gradual during the period immediately preceding the convulsion and would, therefore, account for the "diffused" auras which are so indefinite as often to escape notice. (6) The absorption of the surplus of this fluid is probably facilitated by the relaxation occurring during the period of unconsciousness, and if this does not occur the irritant remains and status epilepticus results. (7) The increase of this fluid is rendered possible probably as a result of lymphatic and excretory inactivity. (8) Toxemia probably aids in the production of a convulsion by diminishing the resistance of the cells of the cortex or by increasing the quantity and irritant powers of the cerebro-spinal fluid. (9) Lastly, heredity is of importance by producing an organism in which circulatory, lymphatic and excretory functions are illy balanced, and containing a brain that is easily irritated and badly nourished.

1106 Main Street.

